# Extending the reach of single-particle cryoEM

**DOI:** 10.1016/j.sbi.2025.103005

**Published:** 2025-03-03

**Authors:** Ardan Patwardhan, Richard Henderson, Christopher J. Russo

**Affiliations:** 1 EMBL- EBI, Cambridge, CB10 1SD, UK; 2 MRC Laboratory of Molecular Biology, Cambridge, CB2 0QH, UK

## Abstract

Molecular structure determination using electron cryomicroscopy (cryoEM) is poised in early 2025 to surpass X-ray crystallography as the most used method for experimentally determining new structures. But the technique has not reached the physical limits set by radiation damage and the signal-to-noise ratio in individual images of molecules. By examining these limits and comparing the number and resolution of structures determined versus molecular weight, we identify opportunities for extending the application of single-particle cryoEM. This will help guide technology development to continue the exponential growth of structural biology.

## Introduction

**Figure 1:**
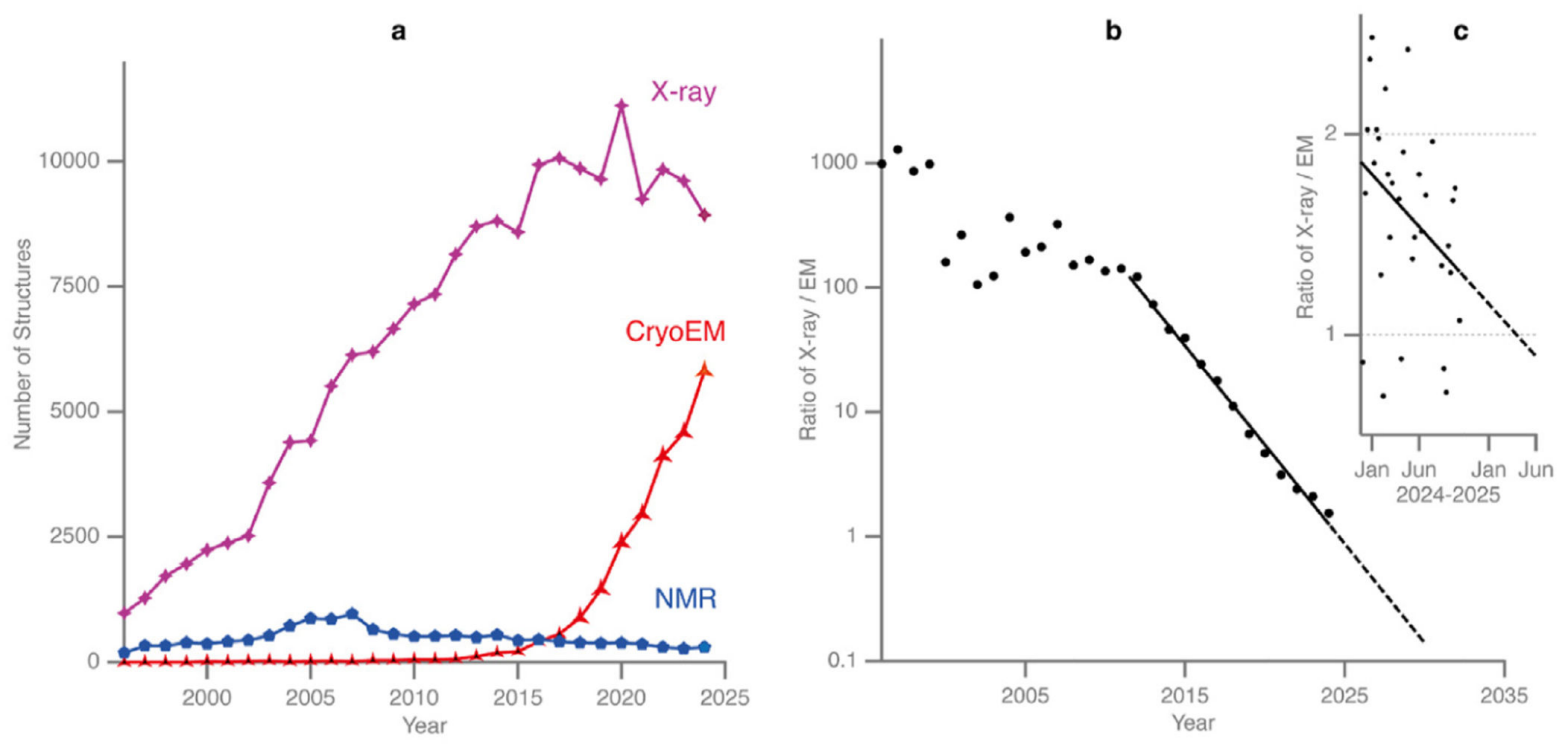
Graphs of the growth in PDB entries categorised by method [X-ray crystallography (magenta), electron microscopy (red) and NMR spectroscopy (blue)] are plotted **(a)**. Points are shown for the number of released PDB entries per annum through the end of 2024. The ratio of X-ray to EM released entries, showing exponential decrease from 2014 until 2022 is also plotted **(b)**, with weekly data shown for 2024 **(c)**, indicating that the date when there will be parity between X-ray and EM entries is expected to be in early 2025.

**Figure 2:**
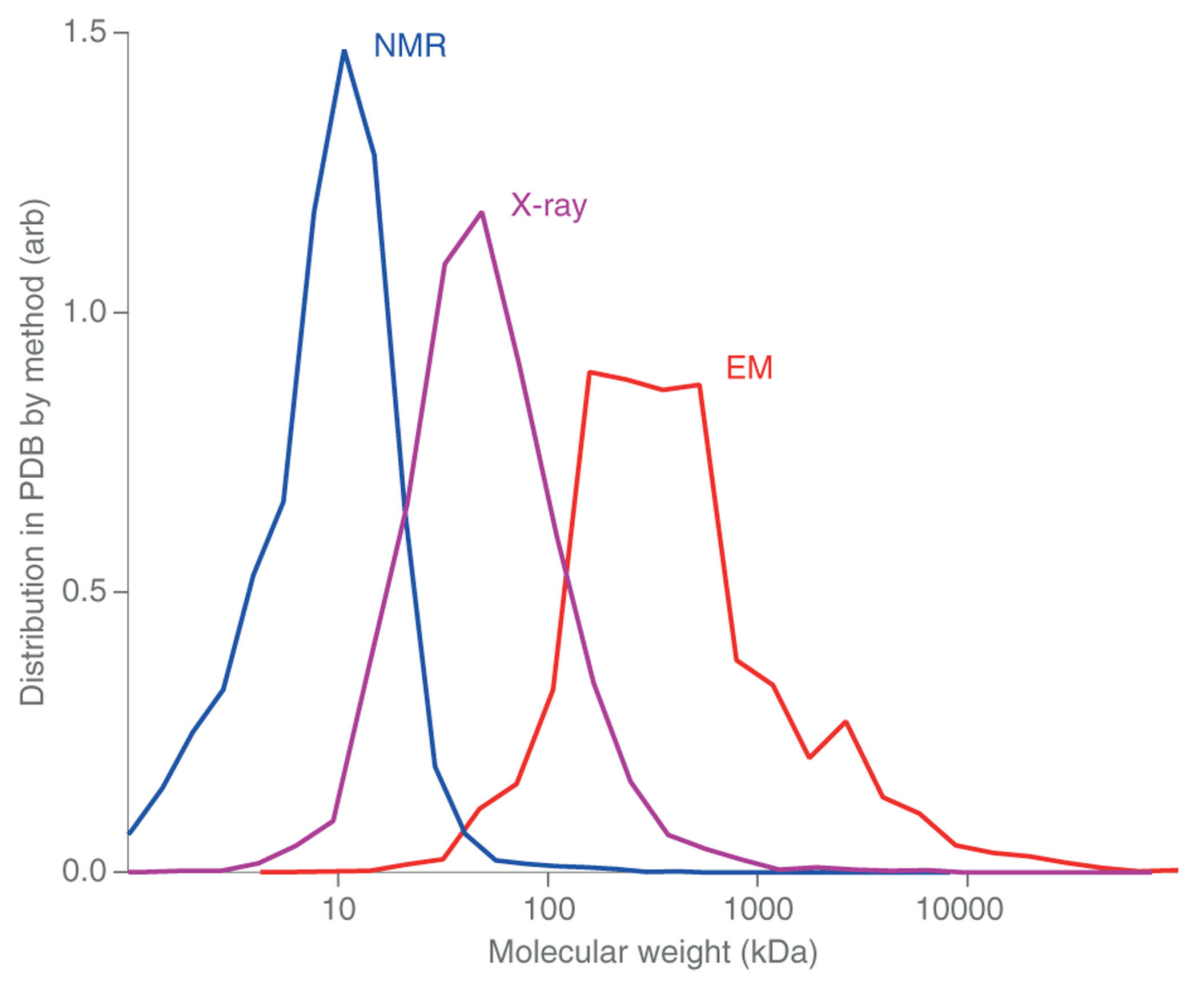
The distribution of structures in the PDB for the three most used methods, NMR, X-ray crystallography and single-particle cryoEM. The histograms are normalized by dividing by the total number of structures determined by each technique, respectively. Each has the same total area.

Single-particle electron cryomicroscopy (cryoEM) has been remarkably successful during the last decade due to improvements in microscopes, detectors and software, as well as advancements in specimen preparation methods (Figure 1). Although there have been theoretical estimates of the expected physical limits on molecular mass and attainable resolution, we asked: what is the best resolution currently attainable with a reasonable effort? By reasonable, we are thinking of one day of data collection or a few thousand micrographs on a state-of-the-art electron cryomicroscope. Of course, it is always possible to reach higher resolution by collecting more (good) images, but beyond one day of data, diminishing returns rapidly set in as the signal used to determine the 3D structure is often limited by factors that decay exponentially with resolution. Existing theory shows that a sufficiently large molecular mass is needed to be able to find the particle orientation from the single-particle electron images whose signal-to-noise ratio is limited by radiation damage. Conversely, large structures have inherent flexibility because biological molecules are soft. Such flexibility is also present in smaller structures but causes less blurring because of the smaller particle size. Together, these two limits set the extents to which cryoEM is amenable to determining any specific structure, given its mass. Even with focused refinement the size of smaller domains that are part of a larger structure is often too small. It is interesting to compare the distribution of cryoEM structure sizes with those determined by X-ray crystallography and NMR (Figure 2).

**Figure 3:**
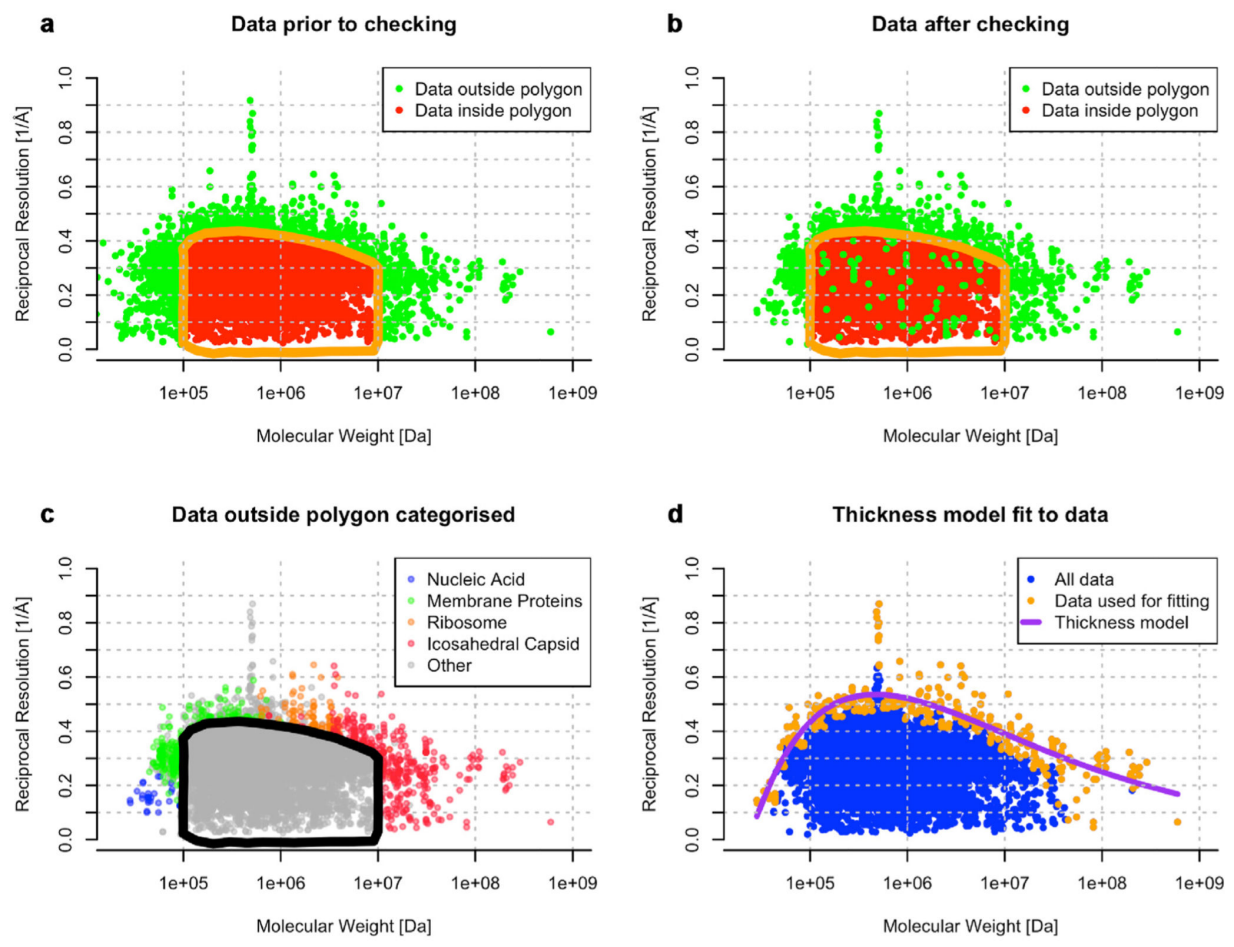
Resolution of structures deposited versus molecular weight, demonstrating the trend for best resolution to be obtained for structures in the 100 kDa to 10 MDa range, with the current optimum around 500 kDa. Data were downloaded from EMDB search on 30th April 2024 of all single-particle entries with fitted models (16,096 entries in total). On checking it was apparent that the molecular weight annotation was not of sufficient quality to allow further analysis without manual triage. For example, molecular weights were sometimes specified for an incorrect assembly or a part of the specimen. It is not possible to automatically derive the molecular weight from the fitted model as they may not always comprise the full specimen. Another complication is that there are many structures in EMDB that are focused/local refinement structures or composite/component structures and are not labelled as such. The manual triage involved correcting molecular weights where possible, e.g., based on information from the related paper, and excluding entries with uncertain annotation, composite structures and parts of specimens. **(a)** Distribution of entries prior to manual triage. To reduce the work effort, we focused on checking entries lying on the outer and upper periphery of the scatter plot, *i.e*., those most likely to affect the trend for best resolution versus molecular weight. A polygon [orange and black outline in panels **(a), (b)** and **(c)**] was hand drawn and only the entries lying outside the polygon were triaged (1897 entries; green) with the rest unchecked (14,199; red). Although the choice of polygon was arbitrary, a comparison of panel **(a)** with **(b)** shows that it was sufficiently removed from the periphery so as not to skew the analysis. **(c)** The same data as in **(b)** but color-coded into manually labelled categories: nucleic acid (blue), membrane protein (green), ribosome (orange), viruses with icosahedral capsids (red) and other (grey). The data inside the polygon was not labelled and is also shown in grey. **(d)** Fitting a model to the top edge of the distribution helps provide a simple estimate of the resolution achievable for a given molecular weight using current technology. This estimate may also be useful during data deposition as a “soft validation” to flag up outliers, both exceptional new structures as well as those with errors and data entry issues. Entries were divided into bins on a logarithmic molecular weight scale (one bin for each integer value of 10 x log (MW) and up to a maximum of 10 entries with the highest resolution (orange) from every bin were selected for model fitting. The rest of the data (blue) was not used for modelling. Among the models tested, the best fitting model was $\frac{1}{\text{R}}=\frac{{\text{C}}_{1}\text{ln}\left(\frac{\text{MW}}{C^{2}}\right)}{{\text{MW}}^{\frac{1}{3}}}$ where C_1_~14 Å^−1^Da^1/3^, C_2_ ~ 23 kDa, 1/R is reciprocal resolution and MW is molecular weight. In broad terms, the logarithmic term describes the linear slope at the lower end of the molecular weight scale while the MW^−1/3^ term describes the falloff with increasing molecular weight. To understand the reason for trying a cube root term of the molecular weight, consider the molecular weight of a spherical macromolecule with a diameter D (or thickness) and constant density $\rho :\text{MW}=\frac{\rho \pi {\text{D}}^{3}}{6}\to \text{D}\propto {\text{MW}}^{\frac{1}{3}}.$, i.e., an inverse resolution dependence on the thickness of the sample. In all charts, the vertical line-like scatter of points at approximately 500 kDa corresponds to apoferritin entries. Apoferritin is a stable assembly that forms a hollow sphere with its mass at a high radius to give accurate orientations. It has become a well-established test specimen for methods development in cryoEM.

**Figure 4:**
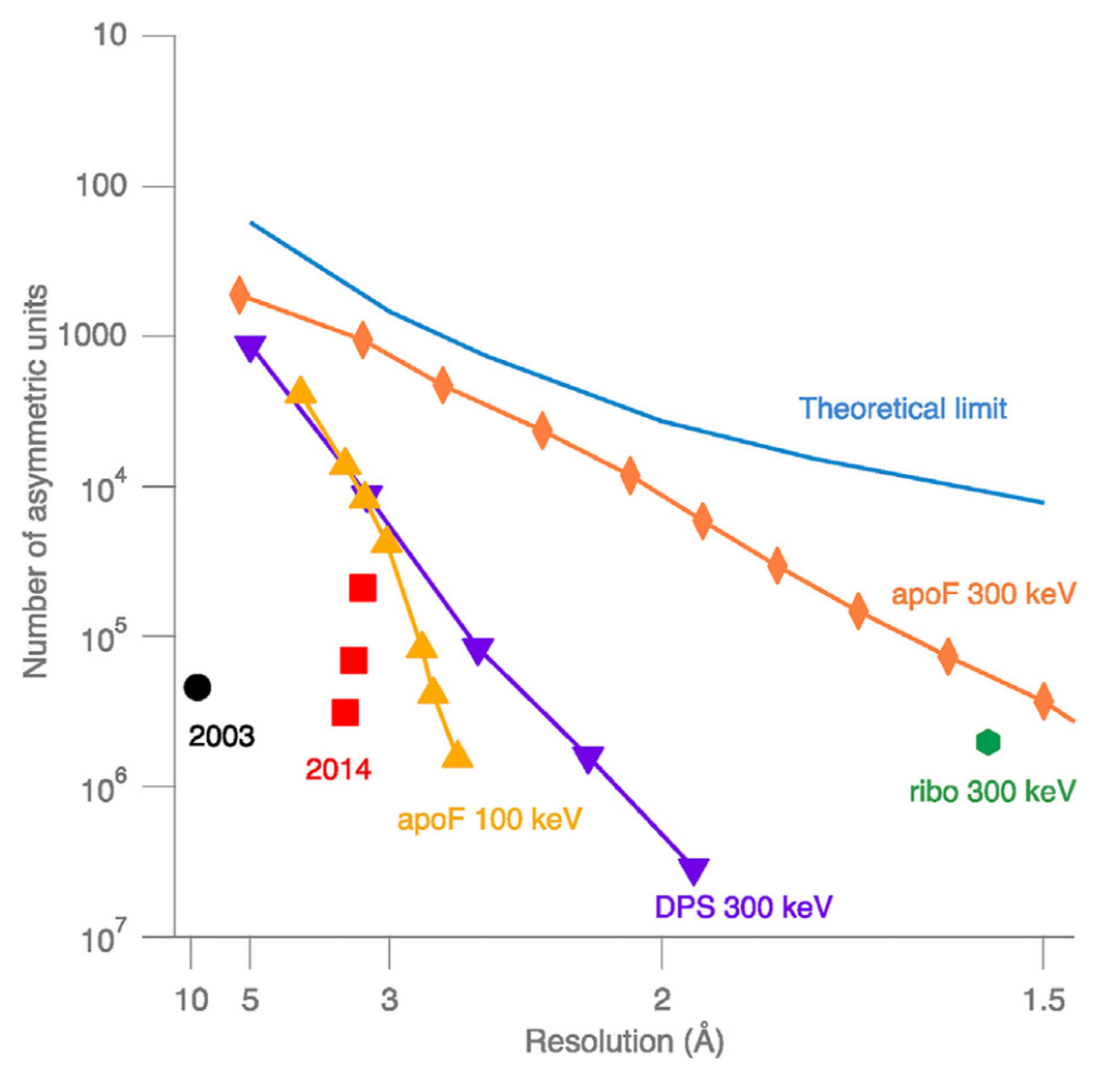
Rosenthal plot of number of asymmetric units (*lnN*) vs. resolution (*1/2d^2^*). The slope of the lines in such a plot determines the effective overall *B*-factor; the reduction in the magnitude of the slope is a measure of the improvement of the technique for structure determination. This shows a comparison of several structures and datasets collected with different technologies over the last decades. Specifically, the first point is from the original plot in Rosenthal and Henderson 2003 [5] (black circle) corresponding to a B-factor for the structure of the icosahedral pyruvate dehydrogenase particle of 1000 Å^2^. The three red squares are the three structures highlighted in the 2014 “resolution revolution” paper [39]. The recent structure of apoferritin determined at 100 keV with an inexpensive cryomicroscope prototype is shown in yellow triangles [36]. DPS (DNA-binding protein from starved cells, blue triangles) [7], apoferritin (orange diamonds) [40], and ribosome (green hexagon) [41] structures determined on high end 300 keV microscopes are also shown. An estimate of the theoretical limit using the theory in Ref. [5] is shown as a blue curve and has an approximate *B*-factor of 20–30 Å^2^. It uses 680 asymmetric units to reach 3 Å resolution together with an extrapolation using the value of N_e_ at different resolutions [10], the formula pD/d for the number of views needed at different resolutions [42], and the theoretical atomic scattering amplitudes for electrons (International Tables Volume C, page 263 [13]).

An analysis of all the three-dimensional (3D) maps in the Electron Microscopy Data Bank (EMDB), and their corresponding atomic models in the Protein Data Bank (PDB), shows these two effects, 1. Sufficient mass to allow enough signal in a single micrograph for particle orientation determination and 2. The inherent flexibility of very large macromolecular complexes. These are borne out in the statistics of all the structures deposited (Figure 3). Finally, the number of images collected to reach a specific resolution is a useful, approximate metric (Figure 4) for quantifying how close current technology is to the physical limits set by radiation damage and electron scattering physics. Given the current state of technology this review attempts to identify how the reach of single-particle cryoEM can be extended to smaller and larger structures. Finally, there is a vast opportunity for improving specimen preparation. Denaturation, concentration of specimens at the airewater interface and preferential orientation continue to plague single-particle cryoEM, which manifests itself statistically in the enormous amount of data that must be discarded to obtain enough good images to solve a structure. Together, these challenges represent the opportunity to extend the reach of cryoEM during the next decade of structural biology.

### Theoretical and physical limits

Radiation damage provides the fundamental constraint on the minimum particle size and attainable resolution for a given number of images or particles. It has been known since the 1970s [1,2] that it is impossible to obtain enough information in a single image of a single molecule to determine its structure before it is destroyed by electron irradiation. Averaging of multiple images is required. For molecules in 3D, those multiple images must also provide a distribution of different views. For structures of identical rigid molecules, estimates of around 1000 images of single-particles to reach around 3 Å resolution have been made. These were based on calculations assuming that all the information from the diffracted electron wavefront can be captured in images recorded on perfect detectors with perfect electron optics [3–6]. The legend to Figure 4 conveys more information about the theory. Because radiation damage causes bond-breakage and rearrangements of the radiolysis products, its effects on the structure can be quite well described by an increase in disorder characterized by a Gaussian function, whose exponent contains a constant, the *B*-factor, that is analogous to a Debye-Waller temperature factor in crystallography. For each incident electron fluence of 1 el/Å^2^ at 300 keV, the overall *B*-factor increases by about 5 Å^2^ [7]. Thus, higher resolution requires more images because the increase in disorder affects the higher resolution Fourier components first, so their signal-to-noise ratio is lower.

Alongside the general increased disorder, there are specific chemical changes and mass losses caused by radiation damage [8,9]. These are due to production and evaporation of the radiolysis products, which are fragments of the initial structure produced by bond breakage. These effects are also dependent on specimen temperature, so that images recorded from specimens at lower temperature retain more information about the original structures. Recent papers [10,11] demonstrate the improvements obtained by lowering the specimen temperature from liquid nitrogen (LN2) at 80K to liquid helium (LHe) at around 13K.

An important physical limit comes from the pseudo-Brownian motion of the water molecules themselves during irradiation. McMullan et al. [12] showed that each water molecule moves by an average of 1 Å for every 1 el/Å^2^ of electron beam irradiation at 300 keV. Also, within about 30 Å of each surface there is loss of hydrogen and oxygen gas and possibly water due to radiolysis of water, which amounts to order of magnitude 0.1e0.2 Å of thinning for every 1 el/Å^2^ of electron beam irradiation at 300 keV.

The form factors that describe electron scattering by atoms are poorly described at present because the scattering factors tabulated in the International Tables Vol C [13]. and used in modelling are based on calculations using neutral, independent, non-bonded atoms. In real structures, especially of biological molecules, all atoms are bonded to other atoms and the electron orbitals shrink. One problem in measuring these form factors experimentally is that the specific effects of radiation damage cause losses, such as decarboxylation of the sidechains of aspartic and glutamic acid. It is possible that determination of structures at much lower electron doses using the latest, movement free specimen supports [14] will allow accurate experimental measurement of form factors for bonded atoms that will also allow the charged state of amino acid side chains in proteins to be observed.

### Experimental and practical limits

Great progress has been made since the 1990s when cryoEM practitioners were termed blobologists, because the resolutions obtained were relatively low. It was not until 1997 that a sub-nm single-particle-structure was obtained [15,16], for hepatitis B virus cores at 7.4 and 9 Å resolutions. Since then, better microscopes, better software and better detectors have made it almost routine to obtain structures in the 2−4 Å region, but there are still many practical and experimental problems that need to be solved.

Microscopes are not perfect so current resolutions and capabilities do not yet reach the theoretical limits set by fundamental physics that have been described above. Finite energy spread in the electron source, combined with the need for adequate space around the specimen that limits the objective lens pole-piece gap and the associated minimum chromatic aberration, gives rise to a temporal coherence envelope function that decreases the signal at high resolution. Although we now have direct electron detectors that are much better than those from 10 years ago [17], they are nowhere near perfect with a Detective Quantum Efficiency (DQE) at Nyquist often in the 20−40 % range. This is the reason that many structures are obtained using quite small pixel sizes of 0.2−0.5 Å which shifts the Fourier components of the structure to lower resolution spacings on the detector. If bigger, faster and higher DQE detectors were developed, this would make an enormous difference to the scale of cryoEM.

All cryoEM maps contain some radiation damage because most cryoEM specimens have received a radiation dose of around 60 MGy (MGy). The specimen, even after only the first frame in a typical cryoEM movie with an exposure of 1 el/Å^2^, has typically adsorbed 3 MGy. Many aspartic and glutamic acid side chains have already been decarboxylated at this dose, and metal centres are known to be affected at even lower doses (0.1 MGy or less). Most practitioners do not mention the implications of this. By comparison, X-ray crystal structures when done carefully often show maps with substantially lower levels of radiation damage because crystals contain a huge number of molecules over which the dose can be distributed so lower doses can be used while still reaching high resolution.

Users need to know how much data they need in practice. The Rosenthal plot shown in Figure 4 is a good way to calculate how much more data is needed to reach a given resolution, but it can only be done for a new specimen once a first 3D map has been successfully made. In practice, the number of particles found in each micrograph frequently deviates greatly from the number expected based on a calculation using Avogadro’s number and the known concentration of the sample. Most specimens are applied at a concentration of 1−10 mg/ml, which is 0.1−1 % by volume, yet the field of view is often (desirably) filled by a close-packed, non-overlapped single layer of molecules. This corresponds to over 100-fold concentration through interaction with the air−water interface. Using the surface of the grid as a concentrator may seem like an alluring technique to obtain structures from proteins that are hard to purify or obtain, but it is often a trap that leads the experimenter into a deep hole requiring enormous amounts of data (and thus money in the form of microscope and computational time) to compensate for the fact that the vast majority of the molecules have been destroyed by interactions with the surfaces present on the grid during concentration.

### Il buono, il brutto, il cattivo

#### Il buono: automation

Less than a decade ago, many of us were collecting datasets on film, one plate at a time, and then scanning each micrograph by hand with the best scanners available. Automation [18], with a lot of help from new direct detectors and software, changed all that. Modern, semi-automated microscopes allow collection of thousands or tens of thousands of micrographs containing millions of individual particle images, helping to increase resolution or make it possible to obtain enough images to determine the structure of rare target particles or rare conformational states. Yet, it also covers up the problem that many of the frozen particles are damaged and lots of the micrographs are bad and would have been immediately discarded if a person was collecting them one at a time. Automation has allowed many good structures to be obtained by essentially a brute-force method where the final structures have been calculated using only a tiny proportion of the observed particles (sometimes 0.1% or less than one particle in the final structure per micrograph). It would be desirable for more work to be targeted at improving the yield of good, well-preserved, non-denatured particles through innovations in specimen preparation. Recent advances in automating the model building process [19], based on new techniques in machine learning, allow atomic models to be built almost instantly given a good 3 Å resolution map. So if the specimen preparation problems can be solved, new rounds of automation development could bring structure determination to levels of sophistication and reliability where DNA sequencing has already been for decades.

### Il brutto: pulsed beams, high-voltage microscopy and 4D STEM

#### Pulsed beams

Some people have advocated using pulsed electron beams for cryoEM, principally because the manufacturers offer this as a possible purchasing option. Preprints have even been published hinting at a potential reduction in radiation damage for cryoEM [20], yet it is difficult to identify any theoretical basis for any difference compared to imaging with a conventional beam. If the average flux is the same in a pulsed and non-pulsed case, then in both situations most events will be separated by about the same time interval. Only a small fraction of the events, in the case of random arrival, will occur significantly closer in time than the average time. How can this relatively small fraction of all events have any significant effect? Even when two events occur much closer in time than the average interval between arrivals, they will almost always occur at sites that are physically far apart. Are we to believe that damage at one point in the specimen is stimulated by damage occurring a short time later, at another point tens of thousands of Ångströms distant? The answer is no, we do not expect any advantage to be obtained from the use of pulsed beams.

#### High-voltage microscopy

Peet et al. [21] showed that for a given thickness of specimen, all other things being equal, there is an optimum energy for imaging biological molecules with electrons, and for the vast majority (>90 %) of specimens this energy is approximately 100 keV. Furthermore, even for thick specimens, there are diminishing returns in increasing the energy of the electron to increase the mean free path since the electron is already travelling at 0.77 times the speed of light at 300 keV. So it is perplexing to us that some recent proposals to build high-voltage electron microscopes for biology have been made. Whilst there are some physical phenomena that have not been accurately measured in the 600 keV to 3 MeV range, like the carbon elastic and inelastic scattering cross sections and critical doses for organic specimens, existing high-energy (MeV) microscopes developed for materials science and installed around the world could be used to explore this regime. Even in theory, microscopes at these energies are bound to yield worse data for most specimens and will cost orders of magnitude more than 100 or 300 keV cryomicroscopes that are currently state of the art.

#### STEM ptychograpy

In materials science, it has been possible to obtain structures beyond the resolution where the electron microscope envelope function is limiting in TEM by using STEM ptychography, because ptychography has the advantage that it bypasses some of the electron-optical limitations of bright-field phase contrast electron microscopy and the specimens are relatively radiation insensitive. Consequently, there has been some enthusiasm to explore application of ptychography to biological structures [22], including simulations of resolutions to be expected once experimental problems have been solved. Recent theory indicates that in the best case, phase recovery by STEM ptychography will be a factor of two worse than perfect bright field phase contrast [23] and recent experimental attempts remain substantially behind standard TEM [24] which is not surprising considering there are many problems like specimen movement that have been solved for TEM but not even approached yet for STEM. Time and experiments will tell if STEM ever catches up to the dominant position that TEM now holds in single-particle cryoEM. Another use of STEM is to perform energy loss spectroscopy during imaging for elemental identification [25].

### Il cattivo: room temperature EM in liquid

#### Room temperature EM

The futility of room temperature electron microscopy of biological molecules in water has been expressed best by Egelman [26] when he wrote a commentary entitled “The myth of high-resolution liquid phase biological electron microscopy”. He explains that a small virus would diffuse by 7 mm during a typical one second exposure, making it impossible to record high resolution images unless much, much brighter sources were developed. In addition, the effects of radiation damage are orders of magnitude greater at room temperature in liquid water than at 100K. For example, for ribosome crystals, the radiation sensitivity at room temperature was found to be 1000x higher at room temperature than at liquid nitrogen temperature [27]. This may depend on the relative stability of the crystals but there is no doubt that the rapid diffusion of radiolysis products including radical species will increase the rate of damage to the molecules at room temperature. Yet papers get published, even in this journal [28], claiming the benefits of room temperature and in-liquid EM for imaging biological molecules but so far, these are misleading.

### Extending the reach

Looking forward, what potential improvements can we expect? The biggest improvements are likely to come through novel methods of specimen preparation. The ultra-small hole gold grids developed to eliminate beaminduced specimen motion [11] are important for specimens at temperatures below 80K, such as 13K, which is near that of liquid helium. Dickerson *et al*. [11] showed that there is a 1.5 times decreased rate of development of disorder due to radiation damage at 13 K compared with 80K, thus allowing longer exposure and an increased signal-to-noise ratio in images. Another recent development is the possibility of trapping time-resolved states by locally melting a frozen sample with a laser beam to trigger protein dynamics in the liquid phase [29]. This might eventually extend the accessible range of time resolution from milliseconds (e.g. Berriman & Unwin, 1994; Subramaniam et al., 1999 [30,31]) to microseconds.

In terms of improvements to be expected from electron optics and lower specimen temperature, it has already been shown that the inelastically scattered electrons contain high-resolution information [6] and that this could be useful, provided that images are recorded close to focus such as by using a Zernicke phase plate to introduce a 90° phase shift in the central unscattered beam. A new type of phase plate based on ponderomotive retardation using a laser confined in a Fabry-Perot cavity offers just such a possibility [32], and overcomes some of the disadvantages of other types of phase plates [33]. Thus, the combination of a laser phase plate with a *C_c_*-corrected electron microscope should improve the information content of images. The combination of liquid helium specimen temperature, chromatic aberration corrected objective lens, and laser phase plate could produce a useful improvement in the quality and information content of images from thicker specimens where a greater proportion of the scattered electrons have lost energy. This may be particularly useful for determining single-particle structures directly from specimens made from cellular lamellae, as was recently demonstrated [34].

To extend the reach of single-particle cryoEM to smaller molecules and complexes in the tens of kDa range, where X-ray crystallography remains dominant, other approaches are needed. The signal-to-noise ratio in individual particle images will need to be improved. Given the theory in Peet *et al*. [21] this may include reducing the energy of the electrons to even lower than 100 keV. Lastly, there is still a desperate need for a good entry-level, affordable cryoEM so that efforts to develop better specimen preparation methods can speed up. This could lead to a faster growth in the field. Efforts in this direction with an emphasis on cryoEM at 100 keV are described in several recent publications [21,35–38]. Taken together, we think the growth of single-particle cryoEM has the potential to continue for another decade or more and the benefits of knowing all these structures will have impact far beyond that.

## Data Availability

All data in this review has been previously published.
